# Biological behavior of preneoplastic conditions of the endometrium: A retrospective 16-year study in south India

**DOI:** 10.4103/0971-5851.65335

**Published:** 2009

**Authors:** Shalinee Rao, Sandhya Sundaram, Raghavan Narasimhan

**Affiliations:** *Jawaharlal Nehru Institute of Postgraduate Medical Education and Research, Puducherry - 605 006, India*; 1 *Department of Pathology, Sri Ramachandra University, Porur, Chennai - 600 116, India*

**Keywords:** *Endometrium*, *outcome*, *preneoplastic*

## Abstract

**Background:**

The biological behavior of endometrial carcinoma differs in epidemiology, presentation, and prognosis, suggesting that there are two fundamentally different pathogenic types of disease: type I (estrogen related, endometrioid type) and type II (non-estrogen related, non-endometrioid type). Untreated hyperplasia can develop into an endometrioid type of adenocarcinoma, hence, it is important to recognize the former type. In contrast to cervical cancers, there are limited studies with respect to the biology of hyperplastic lesions documented from India. This was a 16-year retrospective study, carried out to determine the nature and outcome of proliferative lesions of the endometrium in a referral center from south India.

**Materials and Methods:**

A histopathological diagnosis of the endometrial hyperplasia, polyp, and carcinoma, on endometrial biopsy and hysterectomy specimens, over a 16 year period (1983 to 1999), were recorded in a computer and the case slides were reviewed. Using the computer software Foxpro, the patients who had come more than once for a subsequent or previous biopsy were identified. An attempt was made to look for progression, regression or a static nature of the lesion in the follow-up cases.

**Results:**

A total of 1778 cases were studied, and only 74 patients with endometrial hyperplasia and five cases of benign endometrial polyp had follow-up endometrial histopathology. Hyperplasia cases included 59 cases of simple hyperplasia, 10 cases of complex hyperplasia without atypia, and five cases with atypia. The predominant age for patients with all types of hyperplasias was 41 – 50 years. Progression to a higher grade was seen in 8.10%, regression to a lower grade was seen in 9.45%, lesions reverted to a normal pattern in 10.81% cases, and lesions persisted in 70.27% of the cases. A mixed pattern was seen in 54 cases, with predominant coexistent lesion being simple and complex hyperplasia without atypia.

**Conclusion:**

The fate of the hyperplastic lesion of the endometrium showed a varied pattern. Follow-up cases predominantly showed persistence of the lesion, possibly resulting from a fluctuating but higher level of estrogenic stimulus. Hence, it was not only the high levels of estrogen that influenced the biology, but its sustenance for a prolonged period.

## INTRODUCTION

It was Recamier in 1850, who first recognized the condition of endometrial hyperplasia.[1] Endometrial hyperplasia is the result of a persistent, prolonged, estrogenic stimulation of the endometrium. The most common cause being repeated anovulatory cycles. Hyperplasia may also result from excessive endogenous production or exogenous administration of estrogens.[2] For many years, pathologists have been concerned about the malignant potential of various types of endometrial hyperplasias.

Almost 59 years ago, Hertig and Sommers, proposed the theory of progression of endometrial hyperplasia to adenocarcinoma, going through a stage of atypical changes.[1] Adenomatous and atypical hyperplasias are reported to be the common precursors of endometrial carcinoma.[3] Fox and Buckley drew our attention to the fact that these hyperplastic lesions represent a single disease spectrum.[4] However, most of the studies documented in literature are a reflection of western statistics. The present retrospective study was designed to determine the nature and outcome of proliferative lesions of the endometrium during a 16-year period in a referral hospital in South India.

## MATERIALS AND METHODS

Endometrial biopsies or hysterectomy specimens with diagnosis of endometrial hyperplasia, endometrial polyp, and adenocarcinoma, from 1983 to 1999, were included in the study. The details of patients included in this study were derived from the histopathology requisition files and recorded in a structured proforma, which was then fed into the computer. Using the Foxpro software, patients with more than one endometrial biopsy or who had subsequent hysterectomy were identified. The histopathological status of the endometrium was reviewed and correlated with the clinical history and an attempt was made to look for progression, regression or a static nature of the lesion.

The endometrial hyperplasias were classified according to the World Health Organization (WHO) classification, as simple hyperplasia without atypia (SH), simple hyperplasia with atypia (SHA), complex hyperplasia without atypia (CH), and complex hyperplasia with atypia (CHA).

## RESULTS

**Figure 1:**
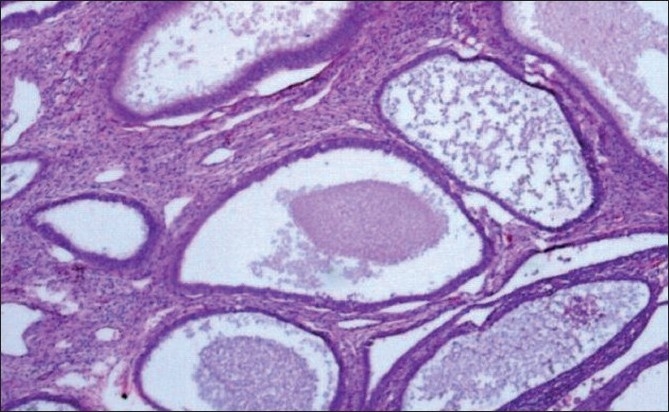
Cystically dilated endometrial glands lined by a single layer of columnar epithelium (Hematoxylin and eosin stain, ×20)

**Figure 2:**
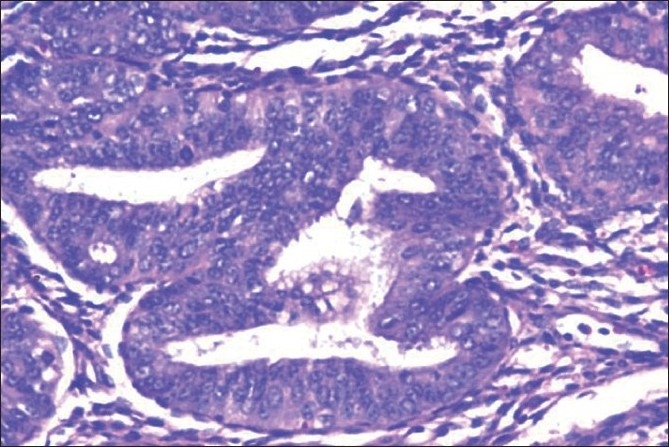
Closely packed endometrial glands with sparse intervening stroma and stratification of the lining epithelium. Epithelial cells show cytological atypia with high nucleocytoplasmic ratio, irregular clumping of nuclear chromatin, and mitotic figures (Hematoxylin and eosin stain, ×200)

A total of 1778 cases of endometrial hyperplasia / polyp / carcinoma were studied. Out of these only 74 patients with endometrial hyperplasia and five cases of benign endometrial polyps came for subsequent follow-up. Patient with endometrial hyperplasia under follow up included 59 cases of SH [Figure 1], 10 cases of CH, and five cases of CHA [Figure 2] at their first visit [Table 1]. The overall follow-up of the study cases showed progression, regression, and persistence of lesion [Table 2]. A varied pattern was seen on follow-up of each subtype of hyperplasia and endometrial polyp [Table 3].

**Table 1:** Type of hyperplasias on their first visit

Type of hyperplasia	No. of cases (%)
Simple hyperplasia	59 (79.72)
Simple hyperplasia with atypia	0
Complex hyperplasia without atypia	10 (13.53)
Complex hyperplasia with atypia	5 (6.75)
Total no. of cases	74

**Table 2:** Biological changes in 74 cases of hyperplasias

Endometrial hyperplasia	No. of cases (%)
Progression to higher grade	6(8.10)
Regression to a lower grade	7 (9 45)
Lesion persisted	52 (70.27)
Reverted to normal pattern	8 (10.81)
Could not be assessed (autolysis)	1 (1.35)

**Table 3:** Behavior of endometrial hyperplasias in the study

	Total cases (Nos.)
Simple hyperplasia	(59)
Progression to complex hyperplasia without atypia	3
Progression to complex hyperplasia with atypia	1
Reversion to normal pattern	4
Lesion persisted	50
Could not be evaluated due to autolysis	1
Complex hyperplasia without atypia	(10)
Progression to complex hyperplasia with atypia	1
Lesion persisted	1
Regression to simple hyperplasia	5
Reversion to normal pattern	3
Complex hyperplasia with atypia	(5)
Progression to adenocarcinoma (in one month)	1
Regression to simple hyperplasia	2
Lesion persisted	1
Reversion to normal pattern	1
Benign endometrial polyp	(5)
Simple hyperplasia	1
Lesion persisted	4

Fifty -four cases out of the 1778 cases in this study showed a mixed pattern of lesion [Table 4]. One case of SH showed the same pattern even after five years, and the existence of a benign endometrial polyp was noticed even after 13 years, in one case.

**Table 4:** Mixed pattern of lesions encountered in 54 of 1778 cases of endometrium studied

Mixed pattern of lesions	No. of cases	Percentage
Simple hyperplasia and complex hyperplasia without atypia	16	36.36
Simple hyperplasia and complex hyperplasia with atypia	3	6.81
Simple hyperplasia with benign endometrial polyp	7	15.94
Complex hyperplasia without atypia and complex hyperplasia with atypia	15	34.09
Complex hyperplasia with benign endometrial polyp	5	11.36
Complex hyperplasia without atypia and adenocarcinoma	6	13.63
Complex hyperplasia with atypia and adenocarcinoma	2	4.54
Total no. mixed pattern of cases	54	100

In the study, the age of patients with endometrial hyperplasia ranged from 12 to 80 years. The mean age of the patients with SH was 39.13 years, whereas, for CH it was 39.99 years, and 42.15 years for CHA [Table 5]. The treatment history was not available for study.

**Table 5:** Age distribution in patients with hyperplasias, benign endometrial polyp, and adenocarcinoma

Lesion	Age range (years)	Mean age group (years)	Common age group (years)
Simple hyperplasia	12 – 72	39.13	41 – 50 (37.4)
Complex hyperplasia without atypia	16 – 80	39.99	41 – 50 (38.8)
Complex hyperplasia with atypia	23 – 69	42.15	41 – 50 (38.6)
Benign endometrial polyp	20 – 65	42.20	31 – 40 (36.9)
			41 – 50 (36.9)
Adenocarcinoma	17 – 84	51.43	41 – 50 (38.3)

Figures in parenthesis are in percentage

## DISCUSSION

Endometrial carcinoma is the most common malignant tumor of the female genital tract in developed countries. The differences noted in epidemiology, presentation, and biological behavior of endometrial carcinoma suggest that there are two fundamentally different pathogenic types of the disease: type I (estrogen related, endometrioid type) and type II (non-estrogen related, non-endometrioid type).[5] Type 1 endometrial carcinoma represents an estrogen-related tumor, which usually originates in the setting of the endometrial hyperplasia, has an endometrioid histology, and tends to be biologically indolent.[5]

Patients with hyperplasia have anovulatory menstrual cycles; some of them may be on exogenous estrogen. Although anovulatory cycles occur at menarche and in postmenopausal women, hyperplasia is not usually encountered in younger women, probably because bleeding in menarchial women is seldom evaluated by an endometrial biopsy. In the present study, the minimum age of presentation was a 12-year-old child with SH. She was younger than the one reported in literature.[6] The most common age group was 41 – 50 years in each type of hyperplasia and endometrial polyp. Schroder *et al*. also documented this to be the most common age group for SH.[7] Endometrial hyperplasia occurs most often in older women in the climacteric as they commonly have anovulatory cycles. This is the age group when ovarian function is in transition as evidenced by pronounced fluctuating estrogen production. During the reproductive years, hyperplasia is relatively uncommon. Women who are obese and have polycystic ovarian disease may have hyperplasia, presumably as a result of the peripheral conversion of androstenedione to estrogen in the adipose tissue. Postmenopausal women develop hyperplasia due to an unopposed estrogen hormone replacement therapy.[8]

The relationship between hyperplasia and carcinoma has been an actively debated subject. Several studies have demonstrated a close relationship of endometrial hyperplasia and carcinoma. Gusberg *et al*., in 1961, observed that the cumulative risk of developing endometrial carcinoma for a patient with AH for 9 to 10 years is significantly higher than for women without hyperplasia.[9] Chamlian and Taylor, in a long term study, found that 14% adenomatous and atypical hyperplasias subsequently developed into carcinoma.[10] Another view suggests that endometrial hyperplasia and adenocarcinoma may represent separate expression of endometrial pathology, which may occur side by side, but need not necessarily follow each other.[11] McBride studied cystic hyperplasia for a period ranging up to 24 years and found that less than 0.4% developed carcinoma.[12]

The fate of a hyperplastic lesion of the endometrium in the present study showed a varied pattern. Tabata *et al*. also found a high percentage of regression including cases with features of atypia. However, they found only one case that progressed to adenocarcinoma.[13] Studies done by Yokosuka *et al*. and Kurman *et al*, also showed a higher percentage of regression as compared to ours, this may be due to the reason that most of the patients in their follow up were being treated with progesterone.[14,15] Our study showed regression to a milder lesion in 9.55% of the cases and reversion to normal pattern in 10.81% of the cases. A lower percentage of case regression in our study could be related to an economical background, illiteracy, and lack of compliance to treatment, as this is a referral government hospital with patient representation predominantly from rural areas. In most of our cases the primary lesions persisted on follow up. The persistence of the lesion could perhaps be explained by high level of estrogenic stimulus with possible intervals of fluctuation. The lesion, which had progressed to a higher grade, would have resulted from sustained high levels of estrogen. In our study, the lesion persisted even after five years in case of SH and for 13 years in case of a benign endometrial polyp, a finding that could be due to fluctuating estrogen levels, not allowing the lesion to progress or regress. On the other hand a sustained higher level could have resulted in progression to a higher grade.

Kurman *et al*. 1985, found that an average period of 4.1 years was needed for atypical hyperplasia to develop into carcinoma.[15] About 17 to 25% of women with atypical hyperplasia, diagnosed in curetting, could have a well-differentiated carcinoma in the uterus if a hysterectomy was performed within one month of curettage.[15] One case of a complex hyperplasia with atypia in our study progressed rapidly into carcinoma within a short span of one month. It therefore, needs to be emphasized that prevalence of endometrial carcinoma in patients harboring an atypical hyperplasia is high. Hence, while planning management strategies for women having atypical hyperplasia, clinicians and patients should take into account the considerable rate of the concurrent carcinoma.[16]

The numbers of prospective studies have been small and the true natural history of the disorder could not be observed due to the administration of various forms of therapies. Retrospective studies may on the other hand give a biased and unduly gloomy view of the risk of adenocarcinoma.

Endometrial hyperplasia of whatever type must be considered as a warning sign that an endometrium is noncycling and therefore susceptible to neoplastic events. The mere presence of hyperplasia is not a basis for hysterectomy. However, in general, the more severe the hyperplasia the more likely it is to be followed by invasive carcinoma. Timely treatment can help to provide an environment for the lesion to regress and avoid radical surgeries. Several investigators have found beneficial effects from treating hyperplasia and carcinoma with progesterone. Kistner, treated patients with atypical hyperplasia and patients with carcinoma in situ with progesterone and found that the lesions were reversible and none advanced.[17]

All forms of hyperplasia in the endometrium appear to be the result of excessive stimulation by estrogen, although such an association is somewhat clear for cystic hyperplasia than it is for atypical hyperplasia.[18] It would be expected from this that two foci of hyperplasia may often be seen in the same uterus. It is not surprising, therefore, to find an endometrium that shows widespread changes of cystic hyperplasia with superadded areas of atypical hyperplasia.[4,18] It is debatable whether the latter develops from the former. It is quite possible to suggest that endometrial glands, which have been constantly stimulated by estrogens for sometime may alter their response and eventually proliferate in an atypical rather than a cystic fashion.[4,18]

However, an alternative explanation could be that the two types of hyperplasia develop concurrently; variable effect being the result of the difference in response from one area of the endometrium to another.[18] In this study, there were 54 cases that showed the co-existence of various subtypes of hyperplasia / adenocarcinoma endometrium. This may be because they either express a common basic abnormality of growth or they form a continuous spectrum.[4] The combined lesions predominantly seen in our study were complex and atypical hyperplasias, suggesting a close relationship between them. According to Fox and Buckley, all types of hyperplasia can coexist with each other except adenomatous hyperplasia (now categorized as CH), which is usually seen in isolation.[4] However, in contrast we found a coexistence of CH with SH and atypical hyperplasias.

Endometrial polyps are a common cause of abnormal uterine bleeding. Rarely, is hyperplasia, either a complex or atypical type, identified within a polyp, in a biopsy or polypectomy specimen. Currently, it is not known whether the hyperplasia is likely to be confined to the polyp or also involves a non-polypoid endometrium.[19]

In our study, we had very few cases to assess this possibility, though we found progression to SH in one case, and a mixed pattern with SH and CH in 12 cases. This could draw our attention to the chances that all were related to each other. In a study done by Antunes *et al*, a low prevalence of premalignant and malignant lesions in endometrial polyps were found.[20] Their study also showed that older women and those with postmenopausal bleeding had a greater prevalence of malignancy and in these cases hysteroscopic resection of the endometrial polyps became mandatory.[20] Kelly *et al*. documented in their study that the risk of endometrial hyperplasia in a polyp, concurrently involving a non-polypoid endometrium was significant.[19]

The nature of the disease was such that patient would require several consultations. Although this was a retrospective study for a period of 16 years, there were limited patients who came for subsequent follow up at our center. Hence, there were chances that the traceability of patients had been lost either because they had lost their out-patient identity card and had re-registered at our center with a new identity number or had gone to a different centre for subsequent visits.

## CONCLUSION

The biological behavior of hyperplasia and the coexistence of various subtypes of hyperplasia along with carcinoma suggest the possibility of a single disease spectrum. In our study, a majority of the cases showed persistence of lesions, with regression and progression in a few cases. This could partly be due to either sustained increased levels of estrogen or a fluctuating high and low levels. This highlights that the levels of estrogen could play a major role in influencing the disease biology and outcome. Further studies correlating to the levels of estrogen and disease progression will provide a deeper understanding. An integrated approach needs to be designed in the healthcare delivery system, with a unique identity number, for maintaining traceability and follow-up of cases for treatment and research purposes. Timely recognition, regular follow up and relevant treatment can prevent disease progression.
